# Sarcoplasmic Reticulum-Mitochondria Kissing in Cardiomyocytes: Ca^2+^, ATP, and Undisclosed Secrets

**DOI:** 10.3389/fcell.2020.00532

**Published:** 2020-06-26

**Authors:** Michela Rossini, Riccardo Filadi

**Affiliations:** ^1^Department of Biomedical Sciences, University of Padua, Padua, Italy; ^2^Neuroscience Institute – Italian National Research Council (CNR), Padua, Italy

**Keywords:** sarcoplasmic reticulum, mitochondria, cardiomyocyte, heart, Ca^2+^, ATP, organelle contacts, MCU

## Abstract

In cardiomyocytes, to carry out cell contraction, the distribution, morphology, and dynamic interaction of different cellular organelles are tightly regulated. For instance, the repetitive close apposition between junctional sarcoplasmic reticulum (jSR) and specialized sarcolemma invaginations, called transverse-tubules (TTs), is essential for an efficient excitation-contraction coupling (ECC). Upon an action potential, Ca^2+^ microdomains, generated in synchrony at the interface between TTs and jSR, underlie the prompt increase in cytosolic Ca^2+^ concentration, ultimately responsible for cell contraction during systole. This process requires a considerable amount of energy and the active participation of mitochondria, which encompass ∼30% of the cell volume and represent the major source of ATP in the heart. Importantly, in adult cardiomyocytes, mitochondria are distributed in a highly orderly fashion and strategically juxtaposed with SR. By taking advantage of the vicinity to Ca^2+^ releasing sites, they take up Ca^2+^ and modulate ATP synthesis according to the specific cardiac workload. Interestingly, with respect to SR, a biased, polarized positioning of mitochondrial Ca^2+^ uptake/efflux machineries has been reported, hinting the importance of a strictly regulated mitochondrial Ca^2+^ handling for heart activity. This notion, however, has been questioned by the observation that, in some mouse models, the deficiency of specific molecules, modulating mitochondrial Ca^2+^ dynamics, triggers non-obvious cardiac phenotypes. This review will briefly summarize the physiological significance of SR-mitochondria apposition in cardiomyocytes, as well as the pathological consequences of an altered organelle communication, focusing on Ca^2+^ signaling. We will discuss ongoing debates and propose future research directions.

## Introduction

**FIGURE 1:**
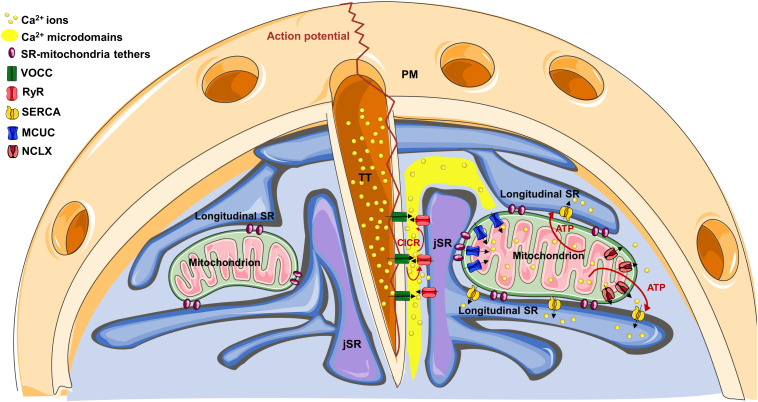
The cartoon represents the structural organization of TT-SR-mitochondria association within a cardiomyocyte. On the right, note how Ca^2+^ microdomains, generated at TT-JSR interface, can reach the edges of juxtaposed mitochondria, favoring mitochondrial Ca^2+^ uptake through the MCUC. See the text for details on the specific Ca^2+^ channel/pump distribution.

In muscle cells, the sarcoplasmic reticulum (SR) is a specialized, differentiated domain of the endoplasmic reticulum (ER), generated from a reorganization of the ER membranes during myogenesis (Michalak and Opas, 2009). SR consists of a network of membranes, closely associated with the myofibrils, specialized in the regulation of Ca^2+^ transport and thus the control of excitation-contraction coupling (ECC). Though ER and SR membranes/lumen are continuous, protein distribution is polarized, with completely different kinetics of Ca^2+^ uptake and release between the two compartments. As an example, ryanodine receptors (RyRs) and calsequestrin are enriched in the SR, whereas inositol-trisphosphate receptors (IP3Rs) and calreticulin in the ER (Michalak and Opas, 2009). Importantly, SR can be divided into two distinct domains: the longitudinal SR (formed by interconnected tubules surrounding myofibrils) and junctional SR (jSR) (formed by the terminal cisternae of the longitudinal SR juxtaposed with TTs) (Figure 1).

As to mitochondria, in adult cardiomyocytes, they are densely packed between jSR and parallel tubules of longitudinal SR, tethered to both SR domains. Specifically, seminal ultrastructural studies revealed, by electron microscopy (EM), a highly orderly organelle juxtaposition between SR and mitochondria, maintained by electron-dense filamentous tethers, whose length is approximately 10 nm (Ramesh et al., 1998; Sharma et al., 2000; Boncompagni et al., 2009; Hayashi et al., 2009). The molecular identity of these tethers is not completely clear (see also below). However, ER-mitochondria “bridges” in non-cardiac cells have been reported to be composed by proteins, to extend for 6–15 nm and to frequently occur in clusters, where they are spaced 13–22 nm apart (Csordás et al., 2006). Importantly, in mice, SR-mitochondria association is generated after birth in a developmentally regulated process, in which mitochondria progressively migrate from a random, predominantly sub-sarcolemma distribution, to their definitive positioning between the myofibrils. In this process, which parallels the maturation of both the primitive TT system and of SR, mitochondria gradually tether to jSR (Franzini-Armstrong, 2007; Boncompagni et al., 2009), with a progressive increase in the occurrence of tethers and organelle contacts.

In the early postnatal days, SR-mitochondria contacts are scarcely observed, possibly suggesting a non-essential role for cell viability; yet, their developmentally regulated appearance hints a structural and/or functional advantage. However, while the ultrastructural features of this coupling have been well defined in the heart, on the other hand (and in contrast with other tissues) its functional significance is still debated. Below, we briefly review these aspects.

## Structural Organization of SR-Mitochondria Coupling

Within SR, the specific distribution of the Ca^2+^ releasing channels (RyR2 in cardiomyocytes) and the sarco/endoplasmic reticulum Ca^2+^-ATPase (SERCA) argues against a significant contribution of mitochondrial localization to the process of mitochondrial Ca^2+^ uptake (see below). Conversely, some experimental evidence suggests this latter could take advantage of the strategic organelle positioning. Indeed, RyRs do not homogenously distribute in the SR membrane, but are specifically recruited on the jSR subdomain facing the TTs (Figure 1). Here, they are closely associated with the TT-located L-type voltage-gated Ca^2+^ channels (VOCCs), originating the “Ca^2+^ releasing unit” (CRU). In these interface regions (dyadic clefts), TTs and jSR juxtapose with a gap of ∼10–15 nm, where electron-dense structures, representing RyR “feet,” are observed (Eisner et al., 2017). Upon an action potential (AP), TTs guarantee an almost simultaneous spreading of sarcolemma depolarization, inducing Ca^2+^ entry through VOCCs. This Ca^2+^ influx generates local Ca^2+^ microdomains, intense enough to stimulate the Ca^2+^-mediated opening of RyRs, a process called “Ca^2+^-induced-Ca^2+^-release” (CICR). In turn, RyR opening triggers a massive Ca^2+^ release from the SR into the dyadic cleft, promptly spreading in the whole cell and ultimately responsible for cell contraction. Of note, mitochondria are excluded from the dyadic clefts, being tethered to jSR on the opposite side (Sharma et al., 2000). This implies that SR lumen separates RyRs from mitochondria (Figure 1). Therefore, before reaching the outer mitochondrial membrane (OMM), Ca^2+^ must diffuse around the jSR cisternae, dissipating part of the steep concentration gradient generated in the nearest CRU proximity. The distance between RyR feet located at the edge of jSR and the OMM of the closest mitochondria is ∼35–40 nm, whereas Ca^2+^, released in the middle of the dyadic cleft, must cover an additional distance to firstly reach the edge of the junction (∼230 nm), and then the OMM (Sharma et al., 2000). This range of distances is considerably higher than that commonly observed in different, non-muscular cell types, where ER-mitochondria Ca^2+^ tunneling is guaranteed by the very close apposition between the two organelles (∼10–25 nm; Filadi et al., 2017c) and by the strategic interaction between IP3Rs on the ER and voltage-dependent-anion-channels (VDACs) on the OMM (Szabadkai et al., 2006).

Sarco/endoplasmic reticulum Ca^2+^-ATPase is also excluded from the dyadic cleft (Fearnley et al., 2011), but is abundant in the longitudinal SR (which surround mitochondria; Greenstein and Winslow, 2011), promptly taking up Ca^2+^ that reaches these regions and further hindering mitochondrial Ca^2+^ uptake. Of note, the overall cytosolic Ca^2+^ peak reached during an AP is ∼1–3 μM (Fearnley et al., 2011). All these considerations argue against the possibility that, during physiological ECC, mitochondria are exposed to sufficiently high Ca^2+^ concentrations [Ca^2+^] to match the relatively low Ca^2+^ affinity of the mitochondrial Ca^2+^ uniporter complex (MCUC). This latter is endowed with a Kd for Ca^2+^ of ∼15 μM and is composed by the pore-forming subunits (MCU and/or the dominant negative isoform MCUb), by the essential component EMRE (a 10 kDa protein spanning the inner mitochondrial membrane, IMM) and by additional regulatory subunits residing in the mitochondrial intermembrane space (MICU1, MICU2 and, in certain tissues, MICU3; reviewed in Mammucari et al., 2018). However, the peculiar ultrastructure of cardiac organelle juxtaposition may overcome these apparent odds. Indeed, at the dyadic cleft: (a) the very close jSR-TT apposition and the small volume of the cleft (Hayashi et al., 2009), strongly favor lateral Ca^2+^ diffusion along the junction, limiting its radial dispersion; (b) several RyR feet fuel CICR and amplify Ca^2+^ microdomain, up to 100 μM (Fearnley et al., 2011); (c) the absence of SERCA (and, possibly, the local depletion of cytosolic Ca^2+^ buffers) enhance Ca^2+^ spreading; (d) longitudinal SR elements, frequently connecting the two jSR cisternae located at the opposite sides of a TT (Boncompagni et al., 2009), may limit Ca^2+^ diffusion outside of the junction (Figure 1). Thus, the specific arrangement of juxtaposed membranes and Ca^2+^ handling proteins may create a “railway” enhancing Ca^2+^ diffusion over relatively long distances, from the junction to the nearby mitochondria, before further spreading in the whole cell. This would imply an heterogeneous Ca^2+^ uptake among mitochondrial population (Pacher et al., 2002; Lu et al., 2013), with those organelles located in closer apposition with the CRU experiencing the highest Ca^2+^ rises.

Interestingly, along the IMM of cardiac mitochondria, the distribution of both the MCUC and the Na^+^/Ca^2+^ exchanger (NCLX, the major mitochondrial Ca^2+^ efflux mechanism in excitable cells; Palty et al., 2010) is not homogenous. Intriguingly, a strategic positioning of MCUC toward areas juxtaposed with jSR has been reported (De La Fuente et al., 2016), whereas NCLX is actively excluded from these regions, but abundantly expressed in the sub-mitochondrial fraction free of contacts (De La Fuente et al., 2018). This biased positioning of the uptake and efflux machineries has been suggested to enhance the efficiency of the excitation-energetics coupling. Indeed, it favors the generation of mitochondrial Ca^2+^ signals capable of sustaining mitochondrial metabolism (see below), while minimizing the energetic cost (in terms of transient mitochondrial depolarization) inevitably associated with mitochondrial Ca^2+^ uptake (De La Fuente et al., 2018). The mechanisms underlying this sub-mitochondrial protein distribution are unknown. However, in rat heart, it has been observed that the OMM-IMM contact points are aligned with those between jSR and mitochondria (Garcia-Perez et al., 2011). This possibly suggests that the OMM-IMM contacts may work as a platform for the anchorage of specific structures (such as the tethers connecting the OMM with SR, composed by proteins possibly anchored to particular lipids) and the recruitment/exclusion of specific protein complexes (such as MCUC or NCLX).

Overall, the tightly regulated organization of the machineries that control mitochondrial Ca^2+^ dynamics, specifically observed in cardiomyocytes, argues in favor of the key importance of mitochondria-jSR apposition and, more in general, of mitochondrial Ca^2+^ signaling, for heart physiology.

## SR-Mitochondria Tethers

While the composition of the protein complexes mediating ER-mitochondria physical/functional tethering has been intensively studied in non-muscular cells (Filadi et al., 2017c), the molecular identity of SR-mitochondria tethers has been poorly investigated in the heart. Most studies focused on mitofusin-2 (MFN2), an OMM protein (in part present also at the ER membranes) involved in mitochondrial fusion. The role of this protein as a putative ER-mitochondria tether has been a matter of intense debate, suggesting either a pro-tethering (de Brito and Scorrano, 2008; Naon et al., 2016) or an anti-tethering (Cosson et al., 2012; Filadi et al., 2015, 2017b) function. Similarly, in the heart, contradictory results have been obtained. The cardiomyocyte-specific deletion of the *Mfn2* gene after birth has been observed to reduce the length of SR-mitochondria contacts by ∼30%, but the distance between jSR and the OMM was not significantly altered (Chen et al., 2012). In isolated cardiomyocytes from this mouse model, upon isoproterenol exposure, higher cytosolic and lower mitochondrial Ca^2+^ rises, associated with a reduced stimulation of the Krebs cycle, were observed. On the other hand, in a different mouse model in which *Mfn2* was specifically deleted in the embryonic heart, jSR-mitochondria apposition was found to be maintained, without significant differences in Ca^2+^ dynamics (Papanicolaou et al., 2011). Importantly, profound alterations of mitochondrial morphology were observed in both models. However, to the best of our knowledge, *Mfn2* ablation has never been reported to completely disrupt SR-mitochondria contacts, nor to decrease the presence of electron-dense filamentous bridges between the two organelles, possibly suggesting that MFN2 is not essential for their formation. Likely, multiple tethers may exist and exert redundant activities.

For instance, recently, the cardiac-specific downregulation of protein-tyrosine-phosphatase-interacting-protein 51 (PTPIP51) has been demonstrated to reduce the extension of SR-mitochondria contacts, while increasing organelle distance, reducing caffeine-induced SR to mitochondria Ca^2+^ transfer and protecting from ischemia-reperfusion damage (Qiao et al., 2017). The opposite effects have been observed upon PTPIP51 overexpression, suggesting that it may participate in contact stabilization, as reported for ER-mitochondria tethering (Stoica et al., 2014). Similarly, FUN14-domain-containing 1 (FUNDC1) on the OMM has been shown to bind IP3R2 on ER membranes, maintaining structural organelle coupling, favoring IP3-linked ER-mitochondria Ca^2+^ shuttling and sustaining cardiac functionality (Wu et al., 2017). However, whether FUNDC1 activity is limited to ER-mitochondria association, or extend also to the SR-mitochondria one, is not completely clear. Indeed, whether specialized SR-mitochondria tethers exist, or they are completely redundant with those mediating ER-mitochondria apposition, remains an unanswered question.

## Functional Evidence of SR-Mitochondria Ca^2+^ Coupling

As far as SR-mitochondria Ca^2+^ transfer in the heart is concerned, in the microdomain that bathes the ends of the mitochondria closer to the CRUs, mathematical models calculated a [Ca^2+^] of ∼10-20 μM (Williams et al., 2011, 2013), lasting for ∼10 ms (Cheng and Lederer, 2008). In rat neonatal cardiomyocytes, the existence of Ca^2+^ hot-spots during spontaneous Ca^2+^ oscillations has been demonstrated by an OMM-targeted FRET-based Ca^2+^ probe (Drago et al., 2012). Upon caffeine-induced RyR stimulation, a prompt mitochondrial Ca^2+^ uptake was observed in permeabilized rat ventricular cardiomyocytes, with a marginal reduction when the fast Ca^2+^ buffer BAPTA was included in the experimental buffer (Sharma et al., 2000). Similarly, in permeabilized cardiac H9c2 cells stimulated with caffeine, it has been calculated that ∼25% of the Ca^2+^ released from SR is taken up by mitochondria (Pacher et al., 2000), further suggesting that they are exposed to sustained Ca^2+^ microdomains. It is worth noting, however, that caffeine stimulation triggers a higher and slower cytosolic Ca^2+^ rise, compared with the physiological Ca^2+^ oscillations observed during ECC, thereby differently impacting on the machinery that mediate mitochondrial Ca^2+^ uptake.

Whether and how mitochondria decode cytosolic Ca^2+^ transients during ECC is debated. Contrasting data have been reported, suggesting either smoothened, slow changes in steady-state mitochondrial Ca^2+^ levels (Miyata et al., 1991; Griffiths et al., 1997; Sedova et al., 2006), or rapid beat-to-beat oscillations, closely following those in the cytosol (Chacon et al., 1996; Robert et al., 2001; Bell et al., 2006; Maack et al., 2006; Garcia-Perez et al., 2008; Drago et al., 2012). The controversy may depend on both the specific Ca^2+^ probes (chemical dyes or genetically encoded indicators) and types of stimulations (electrical, β-adrenergic or spontaneous contraction) used in the different studies. This topic has been extensively reviewed and the interested readers are referred to a comprehensive contribution (De la Fuente and Sheu, 2019). Recently, however, by using the FRET-based Cameleon Ca^2+^ probe 4mtD3cpv expressed in cultured rat cardiomyocytes, it has been demonstrated that, upon a low frequency electrical stimulation (0.1 Hz), mitochondria promptly take up Ca^2+^ (rise time ∼49 ms), whereas their Ca^2+^ efflux is relatively slow (decay half time ∼1.17 s) (Wüst et al., 2017). At higher frequency stimulation, this asymmetry results in a progressive rise in mitochondrial [Ca^2+^], until a new balance between uptake and efflux is reached, yet maintaining a beat-to-beat oscillatory pattern (Wüst et al., 2017). These results confirmed those previously observed in rabbit ventricular myocytes, where the repetitive rise in mitochondrial [Ca^2+^] was observed to be higher and to occur earlier in the pool of mitochondria closer to jSR (Lu et al., 2013). Importantly, the observation that the efflux kinetics are much slower than the uptake ones implies that beat-to-beat mitochondrial Ca^2+^ oscillations might be more easily observed (and could have a larger physiological relevance) in organisms endowed with lower heart rates, such as humans [heart rate ∼1–1.5 Hz; note the match with the reported half time of mitochondrial Ca^2+^ efflux, ∼1.17 s (Wüst et al., 2017)].

## Physiological Significance of Mitochondrial Ca^2+^ Uptake in Cardiomyocytes

As to the possible significance of mitochondrial Ca^2+^ transients in the heart, they have been consistently suggested to stimulate ATP synthesis, thus matching the increased energy demand upon cell contraction (Yaniv et al., 2010; Dorn and Maack, 2013). Indeed, in the mitochondrial matrix, Ca^2+^ stimulates Krebs cycle, thereby enhancing the synthesis of NADH and FADH_2_, in turn fuelling the electron transport chain (ETC) and, ultimately, ATP synthase (reviewed in Rossi et al., 2019). Moreover, in the mitochondrial-intermembrane-space, Ca^2+^ stimulates the activity of different metabolite transporters, further enhancing mitochondrial metabolism (Rossi et al., 2019). Last, but not least, the Ca^2+^-mediated stimulation of the Krebs cycle increases the mitochondrial antioxidative capacity by enhancing the synthesis of NADPH, in turn essential for the activity of key H_2_O_2_-eliminating enzymes, such as glutathione peroxidase (Kohlhaas et al., 2017). Importantly, reactive oxygen species (ROS, which, in mitochondria, are mainly generated as by-products of oxidative phosphorylation) can act as signaling molecules and regulate the activity of different Ca^2+^ handling protein, including SERCA and RyR2 (Feno et al., 2019). Therefore, a complex crosstalk exists between Ca^2+^ and ROS signaling, in which SR-mitochondria juxtaposition likely plays a key role, as observed for ER-mitochondria interface and IP3R-mediated Ca^2+^ transfer (Booth et al., 2016).

An increase in the heart workload is associated with an increase in the frequency and amplitude of cytosolic Ca^2+^ transients, enhancing myofilament contraction. In turn, this triggers ATP consumption, to allow both muscle relaxation and recovery of basal cytosolic Ca^2+^ levels. Therefore, the capacity of mitochondria to decipher transient cytosolic Ca^2+^ oscillations would represent an elegant mechanism to match, in real-time, the acute ATP demand of the beating heart. On the other hand, slower and smoothened mitochondrial Ca^2+^ dynamics would not provide mitochondria with a sufficient temporal resolution to face acute fluctuations of cell energy need. Interestingly, while the close juxtaposition of mitochondria with jSR favors mitochondrial Ca^2+^ uptake (see above), the organelle coupling with longitudinal SR may provide a structural advantage to fuel SERCA (which is abundant in this SR domain) with both mitochondria-derived ATP (Wilding et al., 2006) and Ca^2+^ released through NCLX after mitochondrial Ca^2+^ uptake (as suggested in different cell models; Malli et al., 2005; Poburko et al., 2009). Overall, evidence has been provided suggesting that mitochondrial Ca^2+^ signals are important for ATP synthesis in cardiomyocytes (Maack et al., 2006; Garcia-Perez et al., 2008; Liu and O’Rourke, 2009; Chen et al., 2012; Bertero and Maack, 2018; Wescott et al., 2019), but whether this regulation is carried out on a beat-to-beat basis is not clear. A defective SR-mitochondria coupling, reducing organelle Ca^2+^ exchange and maximal mitochondrial respiration, while increasing mitochondrial ROS, has been reported to associate with heart aging (Fernandez-Sanz et al., 2014). Recently, a critical role for IP3R-mediated (but not for RyR-mediated) SR-mitochondria Ca^2+^ transfer on mitochondrial ATP synthesis has been reported in mouse ventricular myocytes (Seidlmayer et al., 2016), suggesting that different Ca^2+^ signaling pathways may differently impact on mitochondrial metabolism.

In addition to the role played in the modulation of cardiomyocyte bioenergetics, mitochondrial Ca^2+^ uptake has been suggested to buffer part of the Ca^2+^ released from SR during ECC, thereby shaping cytosolic Ca^2+^ oscillations and modulating cell contraction. In rat neonatal cardiomyocytes, MCU downregulation by siRNA (decreasing mitochondrial Ca^2+^ uptake) increases cytosolic Ca^2+^ peaks and contraction during spontaneous Ca^2+^ pacing, whereas MCU overexpression triggers the opposite effects (Drago et al., 2012). Similarly, in adult cardiomyocytes from an MCU-cKO mouse model, isoproterenol-induced cytosolic Ca^2+^ transients were higher compared with WT animals (Luongo et al., 2015). Finally, upon field stimulation, higher diastolic and systolic cytosolic [Ca^2+^], associated with lower ATP levels, were observed in ventricular myocytes from transgenic mice selectively expressing in myocardium a dominant-negative form of MCU (Rasmussen et al., 2015). However, contradictory results are present in literature. Indeed, earlier studies failed to detect a significant impact of mitochondrial Ca^2+^ uptake on cytosolic Ca^2+^ transients in cardiomyocytes (Andrienko et al., 2009; Williams et al., 2013; Boyman et al., 2014). As discussed above, differences between species or experimental setups may underlie part of the discrepancies. Importantly, it is worth noting that cytosolic Ca^2+^ dynamics, among other determinants, are deeply affected by SERCA activity, which controls both the amount of Ca^2+^ stored within SR and the speed/amplitude of Ca^2+^ re-uptake after release. SERCA activity, however, is quite sensitive to ATP availability (Tian and Ingwall, 1996). Therefore, the process of mitochondrial Ca^2+^ uptake may affect cytosolic Ca^2+^ transients not only directly (by buffering the cation), but also indirectly, by modulating ATP availability.

Above, we discussed the importance of mitochondrial Ca^2+^ signaling in cardiomyocytes. However, recent experimental evidence cast doubts on its overall significance. In particular, three different mouse models, in which whole organism or cardiac mitochondrial Ca^2+^ accumulation was severely compromised by manipulating MCUC composition (Pan et al., 2013; Holmström et al., 2015; Kwong et al., 2015; Luongo et al., 2015; Rasmussen et al., 2015; Wu et al., 2015), displayed an almost normal heart functionality at rest. On the other hand, upon β-adrenergic stimulation, a reduced contractile responsiveness, associated with a decreased cardiac performance, was observed (Kwong et al., 2015; Luongo et al., 2015; Rasmussen et al., 2015). These results may suggest that MCU-mediated mitochondrial Ca^2+^ uptake is dispensable for basal cardiac activity, but is necessary upon acute increases of heart workload. The lack of gross alterations in the absence of MCU might be surprising. Importantly, however, the impact of mitochondrial Ca^2+^ uptake on heart performance could be species-specific, depending on the different heart rates and thus on the amplitude of the beat-to-beat Ca^2+^ oscillations in the mitochondrial matrix (see above). Moreover, it should be noted that, although the process of rapid mitochondrial Ca^2+^ uptake is severely (if not completely) compromised in these models, yet the resting mitochondrial Ca^2+^ levels are not deeply affected (Kwong et al., 2015). This may suggest the possible existence of additional (though less efficient) mitochondrial Ca^2+^ uptake mechanisms. For instance, a mitochondria-specific RyR1 (mRyR1), mediating Ca^2+^ uptake, has been reported to be expressed in the IMM of heart organelles and activated at low cytosolic [Ca^2+^] (Beutner et al., 2001, 2005). Though the existence and the physiological importance of mRyR1 is still controversial, it has been recently suggested to enhance ATP synthesis, by transferring Ca^2+^ from SR to mitochondria upon IP3-dependent cardiomyocyte stimulation (Seidlmayer et al., 2016).

Overall, the importance of a strictly regulated mitochondrial Ca^2+^ signaling is outlined by a series of evidences. NCLX deletion in adult mouse heart is lethal, inducing mitochondrial Ca^2+^ overload, activation of the mitochondrial permeability transition pore, necrotic cell death, and sudden heart failure (Luongo et al., 2017). Importantly, NCLX ablation is well tolerated when performed shortly after birth, hinting unknown compensatory adaptations in the maturing heart, and NCLX overexpression in mice was observed to protect from ischemia-reperfusion injury and reduce infarct size (Luongo et al., 2017). On the same line, in some (but not all) mouse models, MCU deletion has been demonstrated to protect from cardiac ischemia-reperfusion damage, by preventing mitochondrial Ca^2+^ overload (Kwong et al., 2015; Luongo et al., 2015). Moreover, the expression of the dominant negative MCU subunit (MCUb) is particularly abundant in the heart (Raffaello et al., 2013) and MCUC density and activity is very low (Fieni et al., 2012). In particular, MCU translation, regulated by microRNA-1 levels, decreases during postnatal cardiac growth, both in mice and humans, whereas MCUb expression increases in mice (Zaglia et al., 2017). The reason why, during cardiomyocyte maturation, the increase in SR-mitochondria association (Boncompagni et al., 2009) is paralleled by a decreased MCU expression (Zaglia et al., 2017) remains an outstanding question. It is tempting to speculate that, in mature heart cells, mitochondria develop specific toolkits to decipher low-level, basal Ca^2+^ fluctuations, while becoming particularly sensitive to Ca^2+^ overload. Interestingly, in physiologic and pathologic cardiac hypertrophy, MCU protein levels increase, a process prevented by β-blocker treatment (Zaglia et al., 2017). Furthermore, the expression of the MCUC regulatory subunit MICU2 is altered in patients with ventricular hypertrophy, and MICU2-KO mice display diastolic dysfunction, possibly associated with delayed cytosolic Ca^2+^ re-uptake and decreased cardiomyocyte relaxation (Bick et al., 2017).

Finally, in cardiomyocytes, cyclic AMP (cAMP) and Ca^2+^ signaling are well known to intersect and modulate with each other (reviewed in Filadi et al., 2017a). Interestingly, recently, in neonatal rat ventricular cardiomyocytes, the presence of a compartmentalized cAMP-PKA signal at the OMM has been demonstrated (Burdyga et al., 2018). Whether the narrow gap between SR and OMM might further shape this localized pathway will require further investigations.

## Conclusion

In the last decade, the rapid growth in our knowledge of the mechanisms regulating the complex balance between mitochondrial Ca^2+^ signal and cell metabolism has tremendously increased our understanding of cardiomyocyte physiopathology, yet prompting additional and unpredicted questions. The intimate relationship between SR and mitochondria appears critical to regulate mitochondrial Ca^2+^ uptake. Nevertheless, whether mitochondria take advantage of their strategic positioning to efficiently detect Ca^2+^ microdomains on a beat-to-beat basis is still matter of debate. Species-specific differences, partly due to diverse heart rates, may dramatically impact on the complex balance between Ca^2+^ uptake/efflux dynamics, resulting in substantially different outcomes. Moreover, the lack of severe phenotypes in mice lacking key MCUC components suggests that additional routes, guaranteeing a sufficient, albeit minimal, Ca^2+^ signal in the mitochondrial matrix might exist. Alternatively, unknown compensatory mechanisms may occur (see for example, the dramatically different effect of NCLX deletion in adult vs newborn mouse heart; Luongo et al., 2017).

In addition to Ca^2+^ transfer, lipid metabolism is known to be modulated by ER-mitochondria tethering; yet, as far as SR-mitochondria juxtaposition is concerned, this aspect has been largely neglected in literature. Similarly, the impact of SR-mitochondria contacts on autophagy, as well as on the regulation of mitochondrial dynamics and morphology, has not been sufficiently explored in the heart. Clearly, an in-depth investigation of the molecules regulating SR-mitochondria coupling appears urgent to study these issues. The development of novel techniques, enabling to simultaneously measure with sufficient spatial and temporal resolution different parameters, will offer the opportunity to further raise the bar, allowing to precisely evaluate the impact of these pathways in different cardiac pathologies.

## Author Contributions

MR and RF revised the literature, wrote and discussed the manuscript. All authors contributed to the article and approved the submitted version.

## Conflict of Interest

The authors declare that the research was conducted in the absence of any commercial or financial relationships that could be construed as a potential conflict of interest.
